# Supplementary material for: Structural Changes Enable Start Codon Recognition by the Eukaryotic Translation Initiation Complex
Source: Cell. 2014 Oct 23;159(3):597–607. doi: 10.1016/j.cell.2014.10.001 (PMC4217140; doi:10.1016/j.cell.2014.10.001)
Supplement: Table S1. Refinement and Model Statistics, Related to Figure 1 [file mmc1.pdf]

Table S1. Refinement and Model Statistics, Related to Figure 1

**Data Collection**	**py48S PIC**	**PIC-2**
Particles	29,698	100,709
Pixel size (Å)	1.34	1.34
Defocus range (μm)	1.6-4.0	1.6-4.0
Voltage (kV)	300	300
Electron dose (e^{-} $Å^{-2}$)	28	28
**Model Composition**		
Non-hydrogen atoms	81,867	77,702
Protein residues	5,389	5,109
RNA bases	1,874	1,778
**Refinement**		
Resolution used for refinement (Å)	4.10	3.85
Map sharpening B-factor (Å)	-52	-88
Average B-factor (Å)	201	189
R factor #	28.9	26.9
Fourier Shell Correlation (FSC)*	0.82	0.85
**Rms deviations**		
Bonds (Å)	0.006	0.007
Angles (°)	1.07	1.18
**Ramachandran plot**		
Favored (%)	82.7	82.5
Outliers (%)	8.0	8.1

Rfactor = $\Sigma||F_{obs}|-||F_{calc}| / \Sigma|F_{obs}|$

* FSC= $\Sigma(N_{shell} FSC_{shell})/ \Sigma(N_{shell})$, where FSC_{shell} is the FSC in a given shell, N_{shell} is the number of "structure factors" in the shell. $FSC_{shell} = \Sigma(F_{model} F_{EM})/ (\sqrt{} (\Sigma(|F|^2_{model})) \sqrt{} (\Sigma(|F|^2_{EM})))$
