# Supplementary material for: Structural Changes Enable Start Codon Recognition by the Eukaryotic Translation Initiation Complex
Source: Cell. 2014 Oct 23;159(3):597–607. doi: 10.1016/j.cell.2014.10.001 (PMC4217140; doi:10.1016/j.cell.2014.10.001)
Supplement: Document S1. Article plus Supplemental Information [file mmc3.pdf]

Structural Changes Enable Start Codon Recognition by the Eukaryotic Translation Initiation Complex

Tanweer Hussain,[1,5] Jose L. Llácer,[1,5] Israel S. Fernández,[1] Antonio Munoz,[2,4] Pilar Martin-Marcos,[3] Christos G. Savva,[1] Jon R. Lorsch,[2,4,6] Alan G. Hinnebusch,[3,6] and V. Ramakrishnan[1,*]
[1]MRC Laboratory of Molecular Biology, Cambridge CB2 0QH, UK
[2]Department of Biophysics and Biophysical Chemistry, Johns Hopkins University School of Medicine, Baltimore, MD 21205, USA
[3]Laboratory of Gene Regulation and Development, Eunice K. Shriver National Institute of Child Health and Human Development, National Institutes of Health, Bethesda, MD 20892, USA
[4]Laboratory on the Mechanism and Regulation of Protein Synthesis, Eunice K. Shriver National Institute of Child Health and Human Development, National Institutes of Health, Bethesda, MD 20892, USA
[5]Co-first author
[6]Co-senior author
*Correspondence: ramak@mrc-lmb.cam.ac.uk
http://dx.doi.org/10.1016/j.cell.2014.10.001
This is an open access article under the CC BY license (http://creativecommons.org/licenses/by/3.0/).

SUMMARY

During eukaryotic translation initiation, initiator tRNA does not insert fully into the P decoding site on the 40S ribosomal subunit. This conformation (P_{OUT}) is compatible with scanning mRNA for the AUG start codon. Base pairing with AUG is thought to promote isomerization to a more stable conformation (P_{IN}) that arrests scanning and promotes dissociation of eIF1 from the 40S subunit. Here, we present a cryo-EM reconstruction of a yeast preinitiation complex at 4.0 Å resolution with initiator tRNA in the P_{IN} state, prior to eIF1 release. The structure reveals stabilization of the codon-anticodon duplex by the N-terminal tail of eIF1A, changes in the structure of eIF1 likely instrumental in its subsequent release, and changes in the conformation of eIF2. The mRNA traverses the entire mRNA cleft and makes connections to the regulatory domain of eIF2α, eIF1A, and ribosomal elements that allow recognition of context nucleotides surrounding the AUG codon.

INTRODUCTION

Eukaryotic translation initiation involves at least 12 initiation factors (eIFs) (Hinnebusch, 2014). The factors eIF1, eIF1A, and eIF3 bind to the 40S ribosomal subunit and promote recruitment of Met-$tRNA_i$ in a ternary complex (TC) with the GTP-bound form of eIF2, a heterotrimeric GTPase, along with the GTPase-activating protein (GAP) eIF5, to assemble the 43S preinitiation complex (PIC). The 43S PIC subsequently binds the 5′ end of the mRNA, a step promoted by eIF4F, eIF4B, and eIF3, and scans the 5′ untranslated region (UTR) of the mRNA for an AUG codon in suitable sequence context. Recognition of the AUG codon by the anticodon of $tRNA_i$ leads to conversion of eIF2 to its GDP-bound form, release of eIFs, and joining of the 60S subunit to form the elongation-competent 80S ribosome with $tRNA_i$ and the start codon base paired in the 40S P site.

In the current model, eIF1 and eIF1A stabilize an open conformation of the 43S PIC that is compatible with scanning (Pestova and Kolupaeva, 2002; Maag et al., 2006) in which TC is in a metastable state (P_{OUT}) that allows $tRNA_i$ to sample successive triplets entering the P site for complementarity to the anticodon. The unstructured C-terminal tail (CTT) of eIF1A plays a key role in stabilizing this open/P_{OUT} PIC conformation (Saini et al., 2010). The eIF5 stimulates GTP hydrolysis by the γ-subunit of eIF2 in the scanning PIC, but completion of the reaction with release of inorganic phosphate (P_i) is blocked by the presence of eIF1 in the complex. Base pairing of $tRNA_i$ with an AUG triplet evokes a rearrangement of factors in the PIC—including displacement of eIF1 and possibly the eIF1A CTT from their locations near the P site—and movement of the eIF1A CTT toward the GAP domain of eIF5 (Saini et al., 2010; Yu et al., 2009; Nanda et al., 2013). These rearrangements enable dissociation of eIF1 from the 40S subunit (Maag et al., 2005; Cheung et al., 2007; Martin-Marcos et al., 2013), evoking a closed, scanning-arrested conformation of the 40S subunit and P_i release from eIF2-GDP (Algire et al., 2005). The $tRNA_i$ is now bound more tightly to the PIC (Passmore et al., 2007), presumably with the anticodon buried deeper in the P site and base paired with the start codon in a conformation dubbed the P_{IN} state (Saini et al., 2010). The unstructured N-terminal tail (NTT) of eIF1A promotes isomerization from P_{OUT} to P_{IN}, enhancing start codon recognition (Saini et al., 2010), but it is unknown how the NTT functions at the molecular level.

Crystal structures of 40S•eIF1 and 40S·eIF1·eIF1A complexes from *Tetrahymena* (Rabl et al., 2011; Weisser et al., 2013), as well as a mammalian 40S·eIF1·eIF1A complex

(Lomakin and Steitz, 2013), revealed a 40S binding site for eIF1 that would clash with $tRNA_i$ bound to the P site in the canonical P/P orientation observed in elongation complexes. A lower-resolution structure of $tRNA_i$ base paired with AUG in a partial mammalian 48S PIC containing eIF1A, mRNA, and deacylated $tRNA_i$ but lacking eIF2, eIF1, eIF5, eIF3, or eIF4F (Lomakin and Steitz, 2013; hereafter referred to as pm48S) suggested that a clash between eIF1 and $tRNA_i$ bound in the P_{IN} state would be instrumental in disrupting eIF1 interaction with the 40S subunit, leading to eIF1 release from the PIC and subsequent events occurring downstream of AUG recognition. In these structures, the functionally important N- and C-terminal tails of eIF1A were not visible. A cryo-EM reconstruction of a partial mammalian 43S PIC (i.e., lacking mRNA) included eIF3, TC, and RNA helicase Dhx29 but lacked eIF1, eIF1A, and eIF5 (Hashem et al., 2013; referred to as pm43S).

Although these previous structures have shed light on interactions of initiation factors with the 40S subunit and the path of mRNA and orientation of $tRNA_i$, the structure of a PIC complexed with eIF1, eIF1A, and the complete TC, with $tRNA_i$ trapped in the act of AUG recognition and the tails of eIF1A visible, clearly would be of great value.

Here, we present the structure of a partial 48S PIC from yeast (hereafter referred to as py48S) containing eIF1, eIF1A, mRNA, and TC at an overall resolution of 4.0 Å. The structure shows $tRNA_i$ in the P_{IN} state interacting with the AUG start codon of mRNA in the P site and reveals changes in the conformation of eIF1 that are likely involved in triggering its release from the PIC following start codon recognition. It also reveals the NTT of eIF1A and its interactions with the AUG, anticodon, and +4 consensus nucleotide in mRNA. In fact, the entire path of the mRNA in the 40S subunit is visible, identifying multiple interactions with initiation factors or ribosome constituents, including contacts of eIF2α-D1 and uS7 (using the nomenclature of Ban et al., 2014), with the −3 position of mRNA previously identified as important. These structural details account for numerous genetic and biochemical findings underlying the current model for AUG recognition by the scanning PIC.

RESULTS

Formation of a Yeast Partial 48S PIC Intermediate Harboring eIF1 and $tRNA_i$ Bound in the P_{IN} State

We assembled py48S using 40S subunits from the yeast *Kluyveromyces lactis*, initiation factors eIF1, eIF1A, eIF3, eIF5, and TC (consisting of eIF2, GDPCP and Met-$tRNA_i$) from *Saccharomyces cerevisiae*, and an unstructured 25 nucleotide mRNA containing an AUG codon but lacking a 5′ cap. Although eIF4F, eIF4B, and eIF3 are not required for PIC assembly with this model mRNA (Algire et al., 2002), eIF3 was included because it increases the efficiency of PIC assembly (Mitchell et al., 2010). *K. lactis and S. cerevisiae* are closely related species (Figure S1A available online) and *S. cerevisiae* initiation factors form a well-defined PIC with *K. lactis* 40S subunits in vitro (Figure S1B). We used 40S subunits from *K. lactis* because they better tolerate the slightly acidic pH (6.5) chosen to minimize deacylation of Met-$tRNA_i$ (Fernández et al., 2014). To promote formation of a PIC with $tRNA_i$ in the P_{in} state, base paired with AUG, we included eIF5, which has been shown to shift the equilibrium toward this state (Maag et al., 2006; Nanda et al., 2013) and also the U31:A39 variant of $tRNA_i$ (Dong et al., 2014) and Sui3-2 variant of eIF2 (harboring the S264Y substitution in the β-subunit) (Martin-Marcos et al., 2014), which were shown to stabilize the P_{in} state in vivo and in vitro.

Overview of the Structure

The structure of the py48S was determined to an overall resolution of 4.0 Å by single-particle electron cryomicroscopy (cryoEM; Figures 1 and S2 and Table S1). The local resolution and the corresponding density were best in the core of the 40S subunit and for components directly attached to it (Figure S3), where it was possible to see side chains for amino acids (Figure S3C). There is clear density for 40S, eIF1, eIF1A, mRNA, $tRNA_i$, and eIF2α (Figures 1A–1C), which allowed the structures of these components to be modeled and refined. Factors eIF1A and eIF1 are respectively observed in the A site and adjacent to the P site, consistent with previous crystal structures of 40S PICs (Rabl et al., 2011; Weisser et al., 2013; Lomakin and Steitz, 2013). The entire mRNA is visible in the cleft in the 40S subunit and presents the start codon in the P site, where it interacts with the $tRNA_i$. eIF2α is bound in the E site alongside the $tRNA_i$, having a large interface with it. Further away from the 40S platform, eIF2γ is attached to the 3′ end of the $tRNA_i$ acceptor arm (Figures 1A and 1B). The resolution is worse with distance from the 40S (Figures S3A and S3B) probably due to increased mobility. Therefore, no model building or refinement was done for eIF2γ, and its placement (Figure 1D) was based on the structure of the archaeal TC (Schmitt et al., 2012). There is also no interpretable density for eIF2β and eIF5, although as discussed below, we observed additional density that may originate from parts of these factors (Figure 1B, pink). We do not observe density for eIF3, but its inclusion during complex formation resulted in twice as many particles containing TC, consistent with its ability to enhance PIC formation (Mitchell et al., 2010). It may subsequently have dissociated or become disordered.

A distinct class of 40S complexes containing eIF1 and eIF1A (Figures S2 and S4 and Table S1) was used to obtain a map at 3.8 Å. This complex, referred to as PIC-2, contains a mass of density next to eIF1 nearly identical to the one tentatively assigned to eIF5 in py48S (see below). As discussed later, this assignment was based on the size of the density and the known interaction between eIF1 and eIF5-CTD (Asano et al., 2000; Luna et al., 2012). The similarity of PIC-2 to the previous 40S·eIF1·eIF1A crystal structure from *T. thermophila* (Weisser et al., 2013) (Figure S4C) allowed us to build and refine the structure and use it in subsequent analysis. Below, we describe details of the structure and interactions of the various components that make up the py48S.

Initiator tRNA in the Act of Recognizing the AUG Codon

The $tRNA_i$ is anchored deep in the P site in a P_{IN} state with its anticodon base paired to the start codon of the mRNA (Figures 2A and 2B). The overall position of the anticodon stem loop (ASL) of $tRNA_i$ in the P site is similar to that observed in the pm48S with mRNA and eIF1A but lacking eIFs −1 and −2 (Lomakin and Steitz, 2013). However, when compared to $tRNA_i$ present

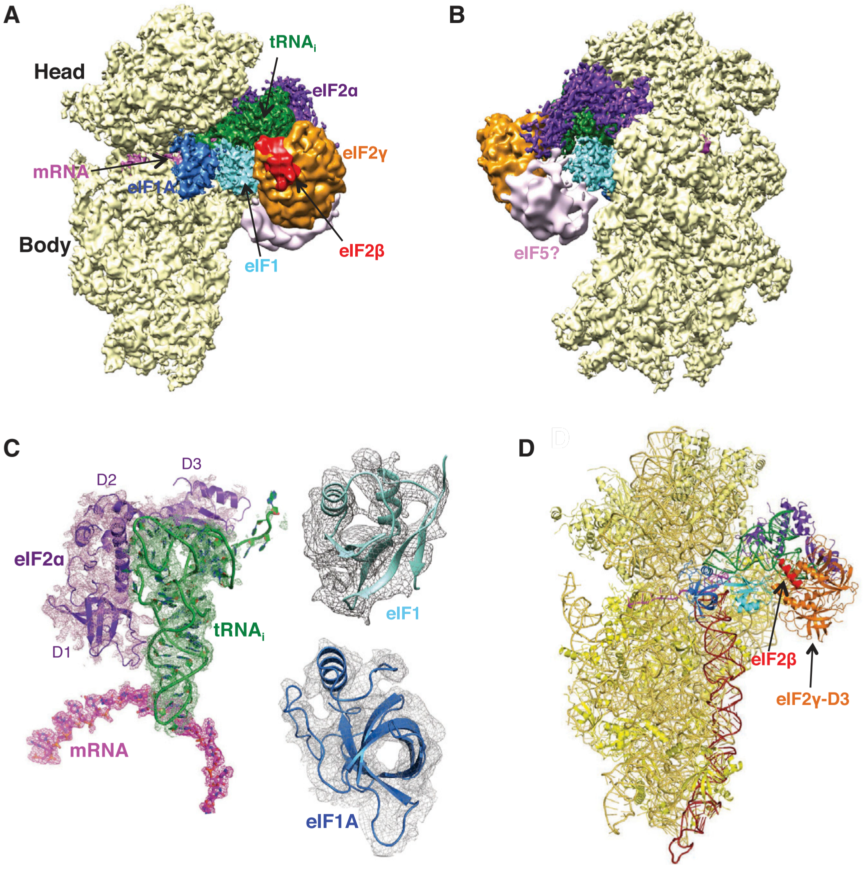

Figure 1. Cryo-EM Structure of the Eukaryotic Preinitiation Complex in the P_{IN} State

(A and B) CryoEM maps of the py48S PIC at 4.0 Å shown in two orientations. Regions of the map are colored by component to show the 40S subunit (yellow), eIF1A (blue), eIF1 (cyan), Met-tRNA$_i^{Met}$ (green), mRNA (magenta), eIF2α (violet), eIF2γ (orange), and eIF2β (red). Density which may correspond to eIF5 is shown in pink. The density for eIF2β, eIF2γ, and eIF5 is low-pass filtered to 8 Å. The same colors are used in all the figures.

(C) Maps at 4.0 Å for tRNA$_i$, mRNA, and eIF2α contoured at 3σ and eIF1 contoured at 2σ.

(D) Atomic model for the PIC in the same colors except that 40S proteins and rRNA are displayed in various shades of yellow. rRNA helix 44 is displayed brown.

See also Figures S1–S4 and Table S1.

in the pm43S (Hashem et al., 2013), the tRNA$_i$ here is positioned ~7 Å deeper into the P site (Figure 2C). This also suggests that, in the absence of mRNA, the tRNA$_i$ in the mammalian 43S structure is in a P_{OUT} state.

The tRNA$_i$ has a different conformation (eP/I′) from the canonical P/P state, the P/I state observed in the pm48S (Lomakin and Steitz, 2013), or the bacterial 30S PIC (Julián et al., 2011) (Figure S5A). Instead, it is similar to the conformation of tRNA$_i$ (eP/I state) observed in the pm43S (Hashem et al., 2013) (Figures 2C and S5A). The acceptor arm is oriented toward the A site, and its 3′ end is displaced upward due to its interaction with eIF2γ. A similar conformation of the 3′ end was observed in an archaeal aIF2 TC (Schmitt et al., 2012). The T loop is displaced toward the E site, possibly due to its interaction with eIF2α and insertion of eIF2α-D1 in the E site. The interaction with eIF2α-D2 slightly distorts the T loop (Figure 2C). The reduced accuracy of initiation caused by a mutation in the T loop (Dong et al., 2014) may thus arise because the distortion is easier to achieve.

A characteristic of tRNA$_i$ is the presence of three conserved G:C base pairs in the ASL. In bacteria, recognition of the minor groove of the first two base pairs by G1338 and A1339 of 16S rRNA was suggested to stabilize the binding of tRNA$_i$ in the P site (Lancaster and Noller, 2005; Qin et al., 2007), as subsequently seen in 70S complexes (Selmer et al., 2006; Korostelev et al., 2006). In the py48S here, these interactions made by the equivalent G1575 and A1576 (*S. cerevisiae* numbering; for the *K. lactis* equivalent, see Figure S1A) of h29 are made possible by both the P_{IN} state of the tRNA$_i$ and a repositioning of h29 (see below) (Figure S5B). Most substitutions of G1575 and A1576 in yeast 18S rRNA are lethal and, in the presence of wild-type rRNA in the same cells, confer a dominant Gcd$^-$ phenotype (Hinnebusch, 2005), indicating impaired TC binding to the PIC and also increased "leaky scanning" wherein an upstream AUG codon is skipped in favor of a start codon further downstream (Dong et al., 2008). Moreover, purine:purine and most pyrimidine:pyrimidine mismatches at the first or second G:C pairs of the yeast tRNA$_i$ ASL are lethal, suggesting that this interaction stabilizes the P_{IN} state (Dong et al., 2014).

It was also shown that disruption of the third G:C base pair destabilizes the P_{IN} state and blocks initiation at non-AUG codons, whereas changing its identity to U31:A39 (Figure S5C) stabilizes P_{IN} and increases initiation at UUG codons (Dong et al., 2014). The conformation of the ASL here and in a previous pm48S PIC (Lomakin and Steitz, 2013) avoids a clash with mRNA and allows base pairing with the codon and differs from that in a 43S PIC (Hashem et al., 2013; Figure S5D). The U31:A39 substitution in the tRNA$_i$ variant used here may make it easier to achieve this conformation, thereby stabilizing the P_{IN} state and allowing imperfect codon-anticodon pairing in vivo.

The Path of mRNA in the 40S Subunit

The mRNA bases at −1 and +4, adjacent to the start codon, are both unstacked from adjacent bases, with the former interacting with G1150 of rRNA and the latter with Trp70 of eIF1A (Figure 2B). These stacking interactions may allow scanning to pause when the start codon is reached. In fact, substitutions in G1150 were shown to confer dominant Gcd$^-$ and leaky-scanning phenotypes, indicating impaired TC binding and AUG recognition in vivo, as were substitutions of C1637, which contacts the +3 nucleotide (Figure 2B; Dong et al., 2008).

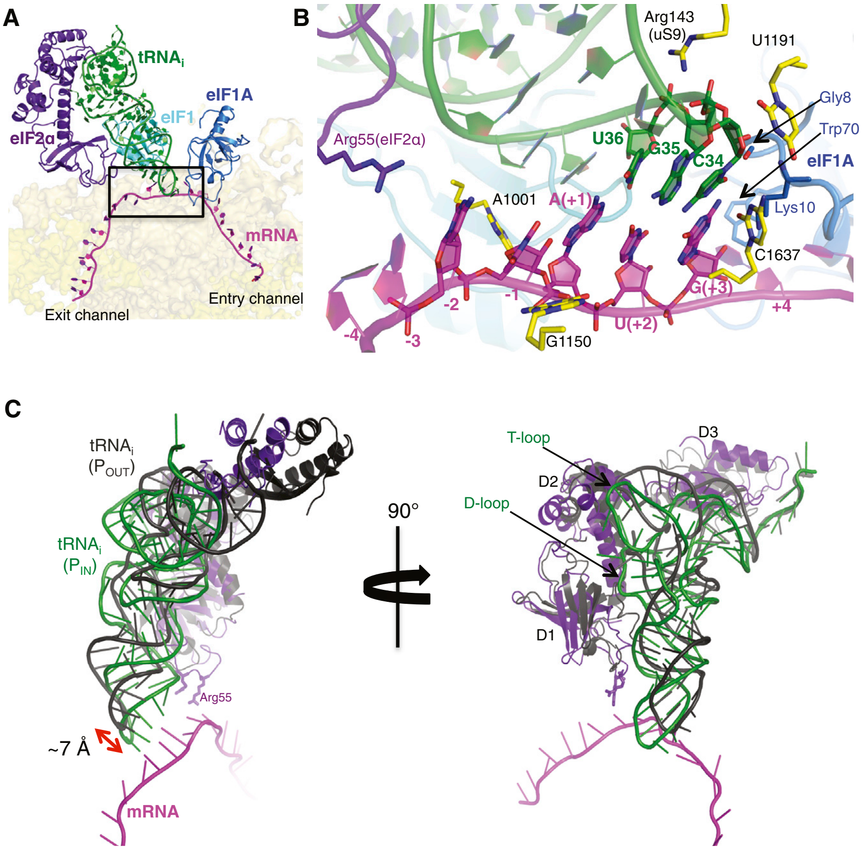

Figure 2. tRNA$_i$ and mRNA: Codon-Anticodon Interaction and Predicted P$_{OUT}$-to-P$_{IN}$ Transition

(A) Cross-section of the 40S subunit showing the tRNA$_i$ in the P site and the mRNA in its channel. eIF1A, eIF1, and eIF2α are also shown.

(B) A detailed view of the codon and anticodon and surrounding elements that stabilize this interaction.

(C) Superposition of the P$_{IN}$ and P$_{OUT}$ (Hashem et al., 2013) ternary complexes. eIF2γ has been omitted for clarity. The tRNA$_i$ in this complex is about 7 Å deeper in the P site than the tRNA$_i$ in the pm43S complex. The eIF2α residues Arg55 and Arg57 are shown as sticks.

See also Figure S5.

The mRNA interacts with elements of the 40S subunit and eIF1A on either side of the start codon (Figure S5E) but makes fewer interactions in the entry channel than in the exit channel. This observation is consistent with the requirement for a minimum 5′ UTR length and the notion that fixing the mRNA in the exit channel is important for efficient AUG recognition, suggesting that the 5′ UTR must be fixed in the 40S exit channel for efficient AUG recognition (Pestova and Kolupaeva, 2002). The mRNA can be seen along most of the 40S cleft (Figure 2A), and 22 of the 25 nucleotides could be modeled. A kink in the mRNA is clearly seen between the P and A sites (Figures 2A, 2B, and S5E), but not as pronounced as in bacteria (Selmer et al., 2006).

A latch or constriction in the mRNA entry channel is formed by interaction between h18 in the body of the 40S and h34 and uS3 in the head. This latch is "closed" in the py48S PIC and PIC-2 complexes (Figure 3A). It was also reported to be closed in previously studied PICs with various combinations of eIF1, eIF1A, mRNA, and tRNA (Weisser et al., 2013; Lomakin and Steitz, 2013). In contrast, an open conformation was seen in a lower-resolution 40S·eIF1·eIF1A complex in which neither eIF1 nor eIF1A was visible (Passmore et al., 2007). In the two structures here, A579 is flipped toward uS3; however, the interactions between them differs among py48S, PIC-2, and the empty 40S (Figure 3A). Similarly, the interaction between h18 and h34 differs among these structures (Figure 3A) because of different positions of the head with respect to the body of the 40S subunit, as discussed below. Thus, although the latch appears "closed" in all structures, the dynamic nature of the interactions may be part of the mechanism that allows mRNA to move through it during initiation.

Rotation of the 40S Head

In the py48S, the head of the 40S subunit is rotated clockwise around h28, which connects it to the body, relative to the empty 40S and PIC-2 structures (Figure 3B). Compared to the empty 40S, the PIC-2 complex displays a head rotation of ~8°, and a further rotation of ~5° occurs in the py48S. The rotation is similar to that previously observed in mammalian complexes (Lomakin and Steitz, 2013). As a consequence of this rotation, h31 avoids a clash with the anticodon of tRNA$_i$, whereas h29 is brought to a position where G1575 and A1576 can interact with the minor groove of the conserved G:C base pairs in the ASL of tRNA$_i$, as discussed earlier (Figures 3C and S5B). The rearrangement of rRNA in the P site observed in the PIC-2 complex may provide a rationale for the improved binding of TC in the presence eIF1 and eIF1A (Figure 3D) (Maag and Lorsch, 2003; Fekete et al., 2007; Cheung et al., 2007) (Hinnebusch, 2014).

The body of the 40S subunit does not show any major conformational changes upon py48S PIC formation when compared to the PIC-2 complex or the crystal structure of the yeast 80S ribosome (Ben-Shem et al., 2011).

Interaction of eIF1A with the Codon-Anticodon Helix

The position of the globular domain of eIF1A in the A site is the same as that observed previously (Weisser et al., 2013; Lomakin and Steitz, 2013) (Figure 4). In both structures here, A1756 of h44 is flipped out to interact with eIF1A (Figures 4B and S6), as seen previously (Weisser et al., 2013).

In the py48S here, all but four residues of the previously unobserved NTT of eIF1A can be traced. Two highly conserved glycines, Gly8-Gly9, allow a sharp turn that permits the tail to loop back after it extends to interact with the tRNA$_i$ and the mRNA in the P site (Figures 4A and 4B). Hydroxyl radical probing had previously suggested an interaction of the NTT with the ASL of

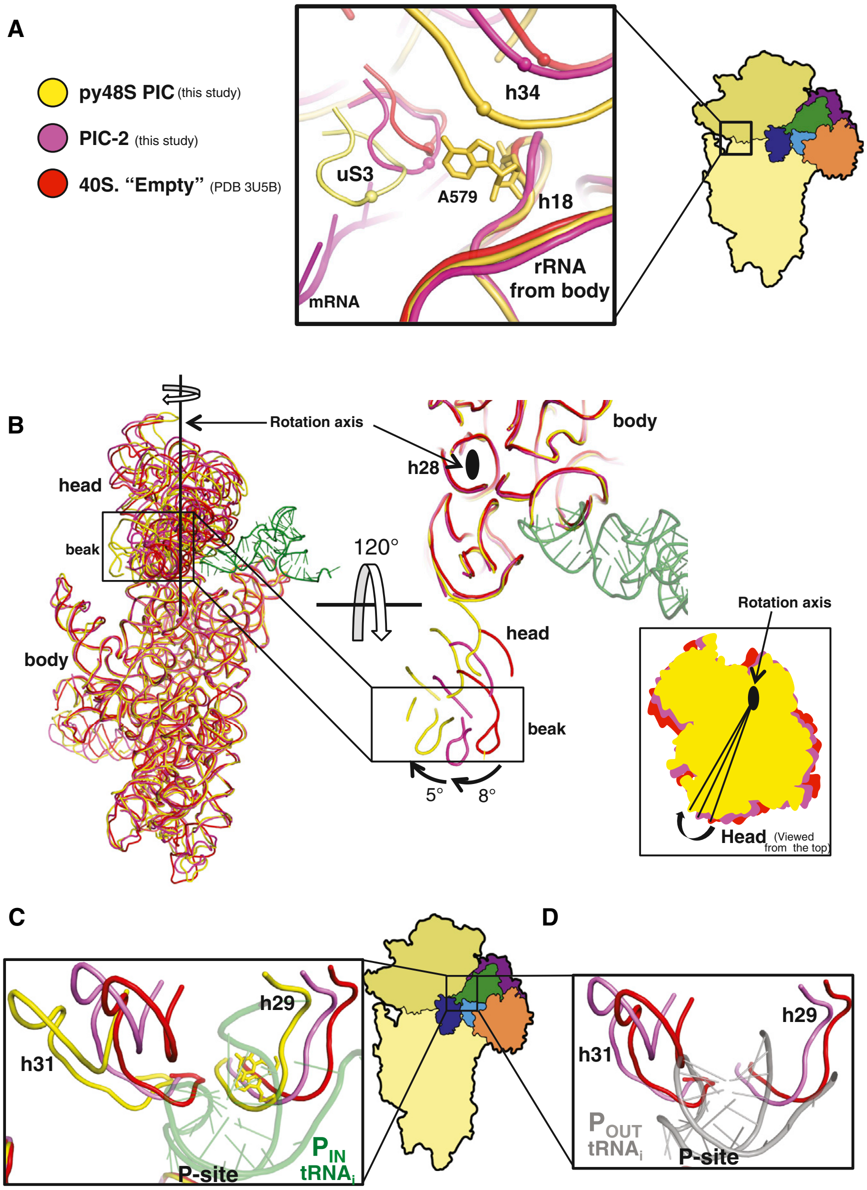

Figure 3. Ribosome Conformational Changes upon Assembly of the Different Preinitiation Complexes

(A) The overall conformation of the mRNA latch is "closed" in the py48S (yellow), the PIC-2 (magenta), and the empty 40S (red; Ben-Shem et al., 2011), but the detailed contacts between the components of the latch are different. Equivalent atoms in h34 and a loop in uS3 are shown as spheres to show the direction of the relative movements in the three structures.

(B) Relative changes in the conformation of the head in the same structures shown in (A).

(C and D) Changes in the environment of the P site resulting from the head movement.

the tRNA$_i$ (Yu et al., 2009). However, those experiments also suggested that the NTT would thread under the tRNA$_i$, contrary to what is observed here. The NTT appears ordered only in the py48S and was not seen in either the PIC-2 complex or in previous PIC structures (Weisser et al., 2013; Lomakin and Steitz, 2013). In the pm48S, additional density proposed to belong to the NTT was observed 7–8 Å away from the ASL (Lomakin and Steitz, 2013), but it was not modeled and did not seem to approach the ASL as observed here.

The loop of the NTT makes interactions with both the anticodon and mRNA and may thereby sense correct base pairing of tRNA$_i$ with the start codon (Figures 2B and 4B). Consistent with these interactions, this segment of the NTT is highly conserved (Weisser et al., 2013). It also interacts with C1637 of rRNA (Figure 4B), consistent with the reduced affinity of eIF1A for the 40S subunit conferred by substitutions in residues 7–11 (Fekete et al., 2007). Immediately downstream of the turn, Arg14 and Lys16 interact with mRNA or the 40S subunit (Figure 4B). These results are consistent with genetic and biochemical findings implicating NTT residues between positions 7 and 21 in promoting the transition from the open/P$_{OUT}$ to closed/P$_{IN}$ states of the PIC for start codon recognition (Fekete et al., 2007; Saini et al., 2010; Luna et al., 2013). Our data thus suggest that direct interactions of the NTT help to stabilize the P$_{IN}$ state.

Trp70 in the globular domain of eIF1A makes stacking interactions with two flipped-out bases, the +4 nucleotide of mRNA and base A1756 of rRNA (Figure 4B). This region is rich in basic residues and makes several other contacts with 18S rRNA, likely explaining why mutations in residues 66–70 of eIF1A weaken its binding to 40S subunits in vitro and in vivo (Fekete et al., 2005).

We do not see a continuous and distinct density for the CTT, making it difficult to interpret its location unambiguously. However, the apparent lack of interaction of the CTT with either eIF1 or tRNA$_i$ would be expected for the P$_{IN}$ state in which eIF1 and the eIF1A CTT have moved apart to accommodate tRNA$_i$ (Yu et al., 2009; Nanda et al., 2013).

Interactions of eIF1 with Initiator tRNA and the Ribosome

eIF1 binds adjacent to the P site with its conserved basic β hairpin loop 1 protruding toward the mRNA cleft at the P site (Figures 4B and 5), as seen previously (Rabl et al., 2011; Weisser et al., 2013; Lomakin and Steitz, 2013). The factor has not been observed before in a PIC containing tRNA$_i$, and its weaker density may arise from lower occupancy, owing to its reduced affinity for the PIC after start-codon recognition (Maag et al.,

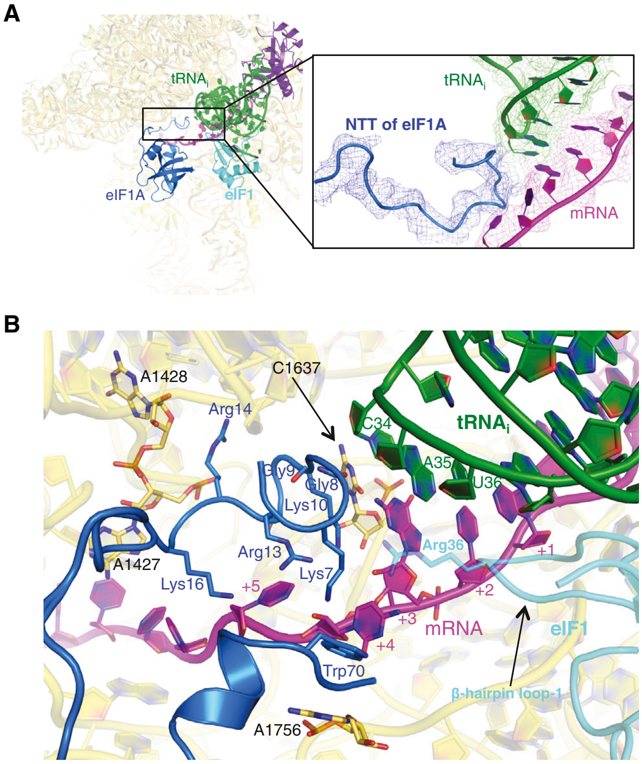

Figure 4. eIF1A in the py48S: NTT Interactions with the Codon:Anticodon Duplex

(A) Fit of eIF1A in the cryoEM map contoured at 3σ (blue). The various PIC components are colored as before. The detail shows the NTT of eIF1A.

(B) Detailed contacts of the NTT of eIF1A with the 40S subunit, $tRNA_i$, and mRNA. The Arg36 of eIF1 (cyan) is also shown as a semitransparent stick.

See also Figure S6 and Movie S1.

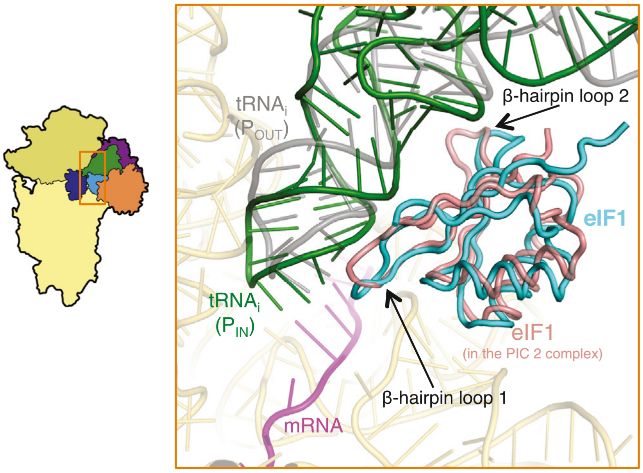

Figure 5. Binding of $tRNA_i$ in the P_{IN} State Perturbs the Structure and Position of eIF1

Comparison of eIF1 in the py48S (cyan) relative to that in the PIC-2 complex (salmon). The two β hairpins of eIF1 are deformed to avoid a clash with the $tRNA_i$ in the P_{IN} conformation of the py48S. By contrast, neither conformation of eIF1 would clash with $tRNA_i$ in the P_{OUT} conformation (gray; Hashem et al., 2013).

2005). Presumably the high concentration of eIF1 used to form the complexes (0.3 μM), which is ~5-fold above the K_d for eIF1 binding to the PIC after start codon recognition (Maag et al., 2005), drove the factor onto the 40S subunit and allowed it to be visualized. It has been suggested that start-codon recognition displaces eIF1 to a new location before it dissociates altogether from the 40S (Nanda et al., 2013). Although we did not observe a large difference in the position of eIF1 between PIC-2 and py48S, the latter exhibits movement of eIF1 β hairpin loops 1 and 2 away from $tRNA_i$, thereby avoiding clashes with the ASL and D-stem (Figure 5). Apart from the loops, the Cα trace of eIF1 from the two complexes also suggests a small displacement of the body of eIF1 in the py48S relative to PIC-2, which may be required to accommodate the altered loop conformations and their interactions with $tRNA_i$. Importantly, the P_{OUT} state deduced from the pm43S (Hashem et al., 2013), in which $tRNA_i$ is not buried as deeply in the P site, would not clash with either conformation of eIF1 (Figure 5), consistent with the fact that eIF1 actually promotes TC binding in the P_{OUT} conformation (Passmore et al., 2007; Martin-Marcos et al., 2013).

The tip of eIF1 loop 1 comes close to the codon-anticodon base pairs, with Arg36 making a direct interaction (Figure 4B). Whereas the interaction of loop 1 with $tRNA_i$ and mRNA is likely to be favorable, loop 2, positioned close to the D-stem backbone of $tRNA_i$ (Figure 5), contains three fairly conserved acidic residues (Asp71, Glu73, Glu76). In the P_{OUT} state, these residues would not be in close contact with $tRNA_i$, whereas a transition to the P_{IN} state would bring them close to the negatively charged phosphate groups of the $tRNA_i$ backbone. The resulting electrostatic repulsion could evoke a change in the conformation of eIF1 and reduce its affinity for the PIC, leading to its eventual dissociation following AUG recognition. In addition, movements of loop 1 produced when the $tRNA_i$ enters the P_{IN} state would disrupt some interactions between this loop and rRNA shown previously to be important for tight binding of eIF1 to the 40S subunit (Martin-Marcos et al., 2013). So eIF1 may promote fidelity by destabilizing the P_{IN} state so that the latter is stable only when the D-stem/loop 2 repulsion is overcome by energy from base pairing of $tRNA_i$ with the AUG codon.

Conformational Changes in eIF2 and Its Interactions with Initiator tRNA

The role of eIF2 in positioning the $tRNA_i$ in the PIC is reflected in the large interface that the three domains of eIF2α have with both $tRNA_i$ and the 40S E site (Figure 6A). Overall, eIF2α occupies the position of an E site tRNA (Figure S7A), with eIF2α-D1 occupying the position of the ASL. eIF2α-D2 mainly forms an interface with the $tRNA_i$ along its anticodon arm, interacting with the D and T loops, whereas eIF2α-D3 forms an interface along the acceptor arm (Figure 6A). Most of the residues forming the interface or interacting with $tRNA_i$ are conserved.

eIF2α-D1 is in a different orientation here than that observed in the crystal structure of the archaeal TC (Figure 6B) (Schmitt et al., 2012). The orientation of the two N-terminal domains (D1 and D2) of the α subunit of eIF2 are rotated and shifted substantially from their positions predicted from the crystal structure of an archaeal

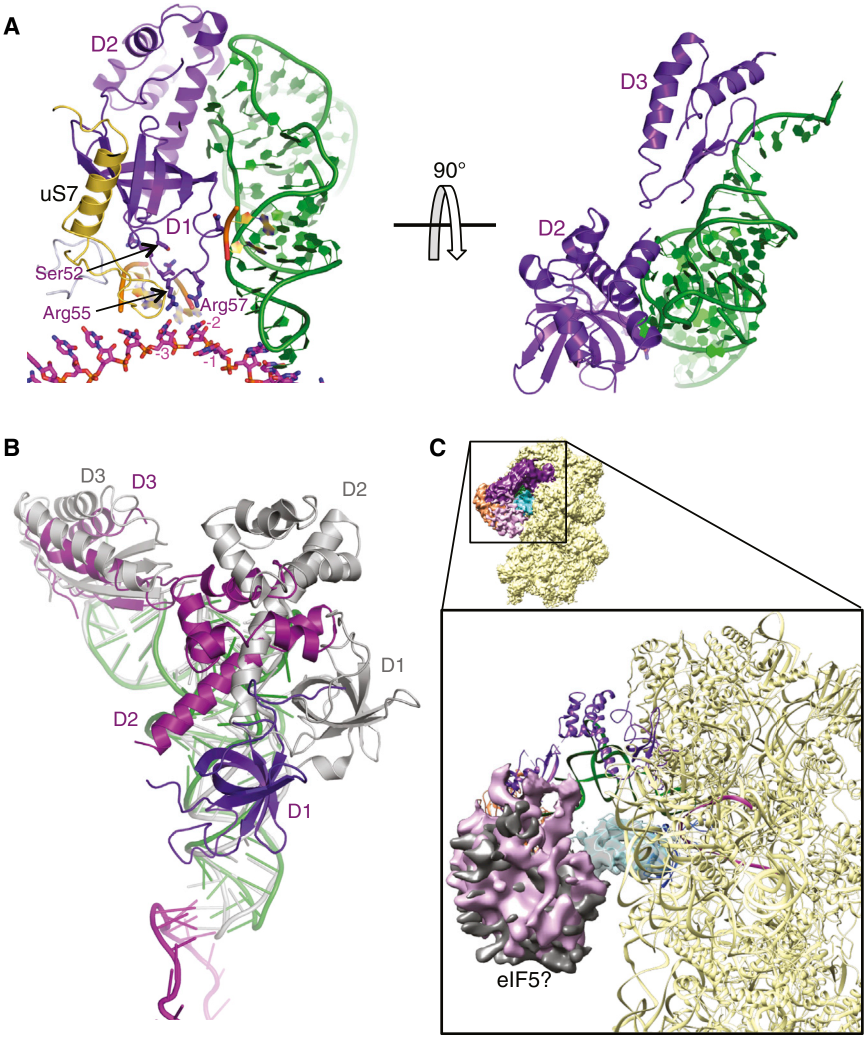

Figure 6. Structural Rearrangement of eIF2α in Formation of the py48S

(A) Interaction of the three domains (D1, D2, and D3) of eIF2α with $tRNA_i$ and mRNA. Arg55 and Arg57 are shown interacting with mRNA at positions −3 and −2. The conserved Ser52 that is the target of phosphorylation is also shown as sticks.
(B) Structural rearrangement of D1 and D2 of eIF2α (shown in different shades of violet) in the py48S compared to their conformations in the isolated aIF2 ternary complex (gray; Schmitt et al., 2012).
(C) Density potentially from eIF5 (pink), connecting eIF1 (cyan), and eIF2γ (orange). Similar density (gray) is found in the PIC-2 complex. Both densities are low-pass filtered to 8 Å.
See also Figure S7.

TC, placing eIF2α-D1 close to ribosomal protein uS7, agreeing with crosslinking data (Sharifulin et al., 2013). This difference in orientations of D1 and D2 was also observed in the pm43S (Hashem et al., 2013). However, the position of eIF2α-D1 is also slightly different than that reported in the pm43S complex, owing to the fact that $tRNA_i$ is inserted deeper into the P site of the py48S, reflecting the difference between P_{IN} and P_{OUT} states (Figure 2C). This in turn places eIF2α-D1 deeper into the E site compared to its location in the pm43S complex (Figure 2C). Importantly, this deeper positioning allows interaction between Arg55 and Arg57 in a conserved loop of eIF2α-D1 and the −3 and −2 positions of mRNA, respectively (Figure 6A), which is consistent with biochemical evidence that interaction of eIF2α with the −3 nucleotide mediates the stimulatory effect of a purine at that position on AUG recognition (Pisarev et al., 2006).

Residue Ser51 (Ser52 in *S. cerevisiae*), in a loop of eIF2α-D1 close to mRNA (Figure 6A), is phosphorylated in a highly conserved mechanism to downregulate general translation initiation in response to various stresses. Phosphorylated eIF2 inhibits its own guanine nucleotide exchange factor, eIF2B, by forming an excessively stable eIF2•eIF2B complex, thereby decreasing the level of active eIF2•GTP in the cell (Hinnebusch et al., 2007). However, there is also some evidence that Ser51 phosphorylation can impair leaky scanning of an upstream AUG codon with suboptimal sequence context (Palam et al., 2011). Based on the structure of the py48S, this could be explained by Ser51 phosphorylation inducing movement of the eIF2α-D1 loop in the mRNA exit channel, which might disrupt either Arg55 interaction with the −3 position of the mRNA or interactions between D1 and $tRNA_i$, reducing the stability of the P_{IN} state and increasing readthrough of start codons.

The conformations of eIF2γ in the py48S and pm43S complex (Hashem et al., 2013) are similar, with eIF2γ-D3 facing, but not interacting with, h44 of 18S rRNA (Figure 1D). The cleavages of h44 directed by Cys residues placed in eIF2γ-D3 (Shin et al., 2011) may thus reflect the unimpeded path of hydroxyl radicals rather than direct contact. With this conformation of eIF2γ and based on either of the two conformations of eIF2β in the structures of archaeal aIF2γβ complexes (PDB codes 3CW2 and 2D74; Stolboushkina et al., 2008; Sokabe et al., 2006), eIF2β would occupy the space between eIF2γ and eIF1A close to the 40S head (Figure S7B).

Putative Location of eIF5

After fitting eIF2 into the observed density, unexplained density remained between eIF2γ and eIF1 closer to 40S platform (Figure 6C). A similar density was present in the same location in the PIC-2 complex. In view of the known interactions of the eIF5-CTD with eIF1 (Asano et al., 2000; Reibarkh et al., 2008), we have tentatively assigned this density to eIF5. Consistent with this assignment, the surface of eIF1 in contact with this density (Figure 6C) is similar to that previously identified by NMR analysis of the eIF1/eIF5-CTD binary complex (Luna et al., 2012).

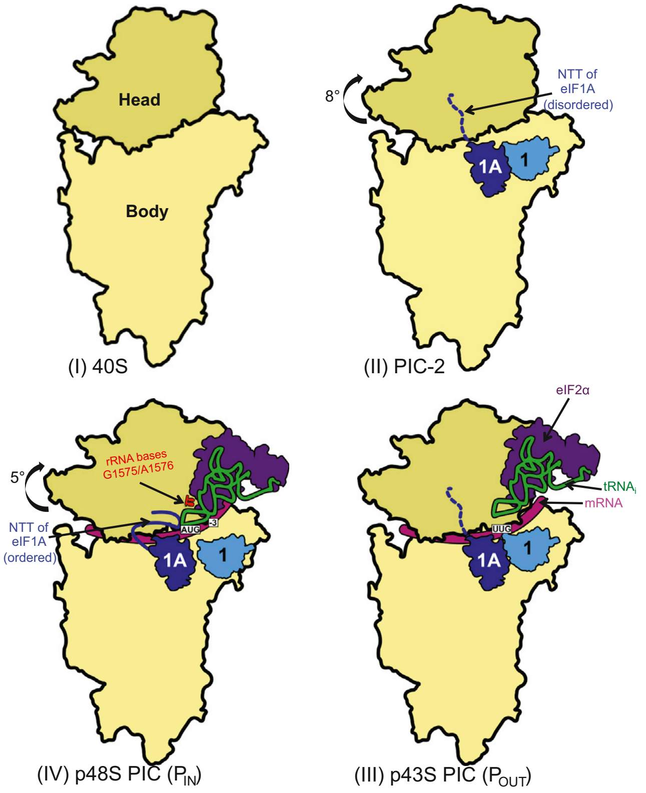

Figure 7. Recognition of the Start Codon in the PIC

Relative to its conformation in the 80S ribosome (I) (Ben-Shem et al., 2011), the head of the 40S in the PIC-2 complex (II) is rotated by 8 degrees. The disordered NTT of eIF1A is shown as a dashed line. The rotation of the head allows TC to bind to the P site in a P_{OUT} conformation as it scans the 5′ UTR for the start codon (III). For clarity, only the eIF2α subunit is shown. Recognition of the start codon is stabilized by the tRNA$_i$ P_{IN} state (IV), which involves a movement of the ASL deeper into the P site. The rRNA G1575/A1576 bases (depicted in red) makes interactions with the minor groove of the conserved G:C pairs in the ASL, the NTT of eIF1A directly engaged with codon:anticodon duplex, and eIF2α-D1 interacting with the −3 position of mRNA in the E site. The head undergoes a further rotation compared to PIC-2 complex.

Considering the ability of eIF5-CTD to promote release of eIF1 from the 40S on AUG recognition (Nanda et al., 2013; Nanda et al., 2009), the py48S here could represent an intermediate state in which the eIF5-CTD remains in contact with eIF1 following the slight displacement of eIF1 from its original location.

DISCUSSION

The structure of the py48S represents a key intermediate of the eukaryotic translation initiation pathway, with the tRNA$_i$ trapped in the P_{IN} state during start-codon recognition (Figure 7). The previously unobserved but highly conserved NTT of eIF1A interacts directly with both the tRNA$_i$ anticodon and the start codon, providing a direct structural basis for its proposed role in stabilizing the P_{IN} state of tRNA$_i$. This P_{IN} state is structurally distinct from the likely P_{OUT} conformation of TC seen in a pm43S complex lacking mRNA, in which tRNA$_i$ is bound less deeply in the P site (Hashem et al., 2013). Together, the two structures illuminate the transition from the P_{OUT} state, poised for transient sampling of codons as they enter the P site, to the P_{IN} state where a stable codon:anticodon duplex is formed at the AUG codon (Saini et al., 2010). We observed that binding of eIF1 and eIF1A to the 40S subunit evokes a rotation of the 40S head by 8° that likely facilitates TC binding in the P_{OUT} conformation. Relative to PIC-2, the transition to P_{IN} at the AUG codon involves an additional 5° head rotation that removes a structural impediment to tRNA$_i$ binding deep in the P site and enables critical A-minor interactions between h29 residues and the conserved G:C pairs of the tRNA$_i$ ASL. We observed that transition to P_{IN} additionally requires a movement in the ASL to permit codon recognition, imposing an energetic penalty that can normally be fully compensated only by a perfect codon:anticodon duplex.

The α subunit of eIF2 structurally mimics E site tRNA, and the loop of its N-terminal domain eIF2α-D1 makes contacts with key nucleotides at the −2 and −3 positions of the mRNA. There is also more extensive interaction of eIF2α-D2 with the ASL of tRNA$_i$, than occurs in the 43S complex (Hashem et al., 2013). These interactions support an important role for the two N-terminal domains of eIF2α in stabilizing tRNA$_i$ in the P_{IN} state and recognizing context nucleotides surrounding the start codon.

Our py48S shows previously unobserved interactions between eIF1 and tRNA$_i$ base paired with AUG in the P_{IN} state. Start-codon recognition triggers dissociation of eIF1 from its canonical location on the 40S subunit, allows for more stable TC binding to the PIC, and is a prerequisite for P_i release from eIF2 (Hinnebusch, 2014). Thus, the py48S here is an important intermediate in the initiation pathway following AUG recognition by tRNA$_i$ but prior to eIF1 dissociation. It is characterized by an altered conformation of eIF1, including deformation of its two β hairpin loops and an adjusted location of the globular domain on the 40S subunit. These changes allow eIF1 to avoid a steric clash with tRNA$_i$ in the P_{IN} state but likely also weaken eIF1 binding to the 40S subunit, leading to its subsequent dissociation.

We proposed that some of the unassigned density observed in the PIC-2 and py48S complexes corresponds to eIF5 and that our py48S might represent an intermediate state in which the eIF5-CTD/eIF1 interaction has not yet provoked eIF1 dissociation from the 40S subunit following isomerization to the P_{IN} state. Distinguishing between the locations of the two eIF5 domains in PICs at different stages of the scanning process and also locating eIF3 and eIF2β within the PIC will have to await additional structures of the 48S PIC in which components more distant from the 40S surface can be visualized in greater detail. Nevertheless, by providing a high-resolution snapshot of the decoding center in a key intermediate during start codon recognition, our py48S reveals the structural basis for much genetic and biochemical data on the roles of tRNA$_i$, eIF1, eIF1A, and eIF2 in the process and provides physical evidence for several conformational transitions proposed to be critically involved in start codon recognition.

EXPERIMENTAL PROCEDURES

Reconstitution of py48S PIC

K. lactis 40S subunits were prepared as described earlier in (Fernández et al., 2014). eIF3 and Sui3-2 mutant eIF2 from *S. cerevisiae* was expressed

in yeast, whereas eIF1, eIF1A, and eIF5 were expressed in *E. coli* as recombinant proteins and purified as described (Acker et al., 2007). Mutant $tRNA_i$ was transcribed and amino-acylated as described (Acker et al., 2007). An unstructured mRNA 25-mer (5′ GGAA$[UC]_4$UAUG$[CU]_4$C 3′) was commercially synthesized by Integrated DNA Technologies. The py48S was reconstituted by incubating 120 nM 40S with eIF1, eIF1A, TC, eIF3, eIF5, and mRNA in the ratio of 40S:eIF1:eIF1A:TC:eIF3:eIF5:mRNA::1:2.5:2.5:1.5:1.2:2.5:2. The sample was directly used to make cryo-EM grids without further purification.

Electron Microscopy

The grids with sample for electron microscopy were prepared as described earlier (Fernández et al., 2014). Data acquisition was done on an FEI Polara G2 microscope operated at 300 kV under low-dose conditions (28 $e^-/\text{Å}^2$) using a defocus range of 1.6–4.0 μm. Images were recorded manually on a back-thinned FEI Falcon II detector at calibrated magnification of 104,478 (yielding a pixel size of 1.34 Å). An in-house system was used to intercept the videos from the detector at a speed of 16 frames/s exposures, as described earlier (Bai et al., 2013). Micrographs that showed noticeable signs of astigmatism or drift were discarded.

Analysis and Structure Determination

We used semi-automated image processing for all reconstructions as described. For the complete data set, 254,957 particles were picked from 1,791 micrographs using EMAN2 (Tang et al., 2007). Contrast transfer function parameters for the micrographs were estimated using CTFFIND3 (Mindell and Grigorieff, 2003). 2D class averaging, 3D classification, and refinements were done using RELION (Scheres, 2012).

Reference-free 2D class averages were calculated to discard defective particles. A total of 244,186 particles were selected for the initial 3D reconstruction using the yeast 40S crystal structure (Ben-Shem et al., 2011) low-pass filtered to 40 Å resolution as an initial reference model. Subsequently, a 3D classification and refinement with fine angular sampling showed that only two classes were homogeneous enough to yield high-resolution reconstructions. The class representing py48S PIC was classified further, and a final class of 29,698 was used to obtain a map at 4.0 Å resolution.

Statistical movie processing was also done as described previously (Bai et al., 2013) in order to improve the resolution of all the reconstructions. The resolutions reported are based on the gold-standard FSC = 0.143 criterion (Scheres and Chen, 2012). Local resolution was estimated using RESMAP (Kucukelbir et al., 2014). All maps were further postprocessed for the modulation transfer function of the detector and then sharpened by applying a negative B factor (-52 for py48S and -88 Å^2 PIC-2) estimated as in (Rosenthal and Henderson, 2003).

Model Building and Refinement

The atomic model of *S. cerevisiae* 40S ribosome (PDBID 3U5B and 3U5C; Ben-Shem et al., 2011) was initially rigid body fitted in density using Chimera (Pettersen et al., 2004), and further model building was done in Coot (Emsley et al., 2010) as described (Fernández et al., 2014). Density for ribosomal protein eL41 was observed in the map and therefore was included in the model.

3D models of eIFs were generated with I-TASSER (Roy et al., 2010). An initial model for the $tRNA_i$ was derived from tRNAs from the archaeal TC (PDB 3V11; Schmitt et al., 2012) and the pm48S (PDB 4KZZ; Lomakin and Steitz, 2013). Model building and refinement were carried out using Coot and Refmac (Murshudov et al., 2011) as recently described (Amunts et al., 2014). All figures were generated using PyMOL (DeLano, 2006) or Chimera (Pettersen et al., 2004).

ACCESSION NUMBERS

Maps have been deposited in the EMDB with accession codes EMD-2763 and EMD-2764 for the py48S PIC and PIC-2, respectively. Atomic coordinates have been deposited in the PDB with accession codes 3J81 (py48S PIC) and 3J80 (PIC-2).

SUPPLEMENTAL INFORMATION

Supplemental information includes seven figures, one table, and one movie and can be found with this article online at http://dx.doi.org/10.1016/j.cell.2014.10.001.

AUTHOR CONTRIBUTIONS

T.H., J.L.L., I.S.F., A.M., and P.M.-M. made the samples. T.H., J.L.L., and C.G.S. collected the data. T.H. and J.L.L. performed the structure determination and analyzed the data. J.R.L., A.G.H., and V.R. supervised the work and helped to write the manuscript.

ACKNOWLEDGMENTS

We are grateful to Shaoxia Chen and Greg McMullan for technical support with cryo-EM, Toby Darling and Jake Grimmett for help with computing, Xiao-Chen Bai and Sjors H.W. Scheres for help and advice with EM data processing, and Garib Murshudov and Alan Brown for help with refinement of the atomic coordinates. We also thank Fan Zhang for providing the purified Sui3-2 mutant of eIF2, Jinsheng Dong for sharing his genetic findings on the U31:A39 $tRNA_i$ variant prior to publication, Ann Kelley and Sarah Walker for providing reagents and advice, and Thomas Dever for his insightful suggestion to employ Sui^- variants. T.H. and J.L.L. were, respectively, supported by postdoctoral fellowships from EMBO and FEBS. This work was funded by grants to V.R. from the UK Medical Research Council (MC_U105184332), Wellcome Trust Senior Investigator award (WT096570), the Agouron Institute and the Jeantet Foundation, from the NIH (GM62128) previously to J.R.L., and from the Human Frontiers in Science Program (RGP-0028/2009) to A.G.H., J.R.L., and V.R., and by the Intramural Research Program of the NIH (A.G.H., P.M.-M., J.R.L., and A.M.).

Received: July 3, 2014
Revised: August 15, 2014
Accepted: October 1, 2014
Published: October 16, 2014

REFERENCES

Acker, M.G., Kolitz, S.E., Mitchell, S.F., Nanda, J.S., and Lorsch, J.R. (2007). Reconstitution of yeast translation initiation. Methods Enzymol. *430*, 111–145.

Algire, M.A., Maag, D., Savio, P., Acker, M.G., Tarun, S.Z.J., Jr., Sachs, A.B., Asano, K., Nielsen, K.H., Olsen, D.S., Phan, L., et al. (2002). Development and characterization of a reconstituted yeast translation initiation system. RNA *8*, 382–397.

Algire, M.A., Maag, D., and Lorsch, J.R. (2005). Pi release from eIF2, not GTP hydrolysis, is the step controlled by start-site selection during eukaryotic translation initiation. Mol. Cell *20*, 251–262.

Amunts, A., Brown, A., Bai, X.C., Llácer, J.L., Hussain, T., Emsley, P., Long, F., Murshudov, G., Scheres, S.H., and Ramakrishnan, V. (2014). Structure of the yeast mitochondrial large ribosomal subunit. Science *343*, 1485–1489.

Asano, K., Clayton, J., Shalev, A., and Hinnebusch, A.G. (2000). A multifactor complex of eukaryotic initiation factors, eIF1, eIF2, eIF3, eIF5, and initiator tRNA(Met) is an important translation initiation intermediate in vivo. Genes Dev. *14*, 2534–2546.

Bai, X.C., Fernandez, I.S., McMullan, G., and Scheres, S.H. (2013). Ribosome structures to near-atomic resolution from thirty thousand cryo-EM particles. Elife *2*, e00461.

Ban, N., Beckmann, R., Cate, J.H., Dinman, J.D., Dragon, F., Ellis, S.R., Lafontaine, D.L., Lindahl, L., Liljas, A., Lipton, J.M., et al. (2014). A new system for naming ribosomal proteins. Curr. Opin. Struct. Biol. *24*, 165–169.

Ben-Shem, A., Garreau de Loubresse, N., Melnikov, S., Jenner, L., Yusupova, G., and Yusupov, M. (2011). The structure of the eukaryotic ribosome at 3.0 Å resolution. Science *334*, 1524–1529.

Cheung, Y.N., Maag, D., Mitchell, S.F., Fekete, C.A., Algire, M.A., Takacs, J.E., Shirokikh, N., Pestova, T., Lorsch, J.R., and Hinnebusch, A.G. (2007). Dissociation of eIF1 from the 40S ribosomal subunit is a key step in start codon selection in vivo. Genes Dev. *21*, 1217–1230.

DeLano, W.L. (2006). The PyMOL Molecular Graphics System. http://www.pymol.org.

Dong, J., Nanda, J.S., Rahman, H., Pruitt, M.R., Shin, B.S., Wong, C.M., Lorsch, J.R., and Hinnebusch, A.G. (2008). Genetic identification of yeast 18S rRNA residues required for efficient recruitment of initiator tRNA(Met) and AUG selection. Genes Dev. *22*, 2242–2255.

Dong, J., Munoz, A., Kolitz, S.E., Saini, A.K., Chiu, W.L., Rahman, H., Lorsch, J.R., and Hinnebusch, A.G. (2014). Conserved residues in yeast initiator tRNA calibrate initiation accuracy by regulating preinitiation complex stability at the start codon. Genes Dev. *28*, 502–520.

Emsley, P., Lohkamp, B., Scott, W.G., and Cowtan, K. (2010). Features and development of Coot. Acta Crystallogr. D Biol. Crystallogr. *66*, 486–501.

Fekete, C.A., Applefield, D.J., Blakely, S.A., Shirokikh, N., Pestova, T., Lorsch, J.R., and Hinnebusch, A.G. (2005). The eIF1A C-terminal domain promotes initiation complex assembly, scanning and AUG selection in vivo. EMBO J. *24*, 3588–3601.

Fekete, C.A., Mitchell, S.F., Cherkasova, V.A., Applefield, D., Algire, M.A., Maag, D., Saini, A.K., Lorsch, J.R., and Hinnebusch, A.G. (2007). N- and C-terminal residues of eIF1A have opposing effects on the fidelity of start codon selection. EMBO J. *26*, 1602–1614.

Fernández, I.S., Bai, X.C., Murshudov, G., Scheres, S.H., and Ramakrishnan, V. (2014). Initiation of translation by cricket paralysis virus IRES requires its translocation in the ribosome. Cell *157*, 823–831.

Hashem, Y., des Georges, A., Dhote, V., Langlois, R., Liao, H.Y., Grassucci, R.A., Hellen, C.U., Pestova, T.V., and Frank, J. (2013). Structure of the mammalian ribosomal 43S preinitiation complex bound to the scanning factor DHX29. Cell *153*, 1108–1119.

Hinnebusch, A.G. (2005). Translational regulation of GCN4 and the general amino acid control of yeast. Annu. Rev. Microbiol. *59*, 407–450.

Hinnebusch, A.G. (2014). The scanning mechanism of eukaryotic translation initiation. Annu. Rev. Biochem. *83*, 779–812.

Hinnebusch, A.G., Dever, T.E., and Asano, K. (2007). Mechanism of translational initiation in the yeast Saccharomyces cerevisiae. In Translational Control in Biology and Medicine, M.B. Matthews, N. Sonenberg, and J.W.B. Hershey, eds. (New York: Cold Spring Harbor Laboratory Press), pp. 225–268.

Julián, P., Milon, P., Agirrezabala, X., Lasso, G., Gil, D., Rodnina, M.V., and Valle, M. (2011). The Cryo-EM structure of a complete 30S translation initiation complex from Escherichia coli. PLoS Biol. *9*, e1001095.

Korostelev, A., Trakhanov, S., Laurberg, M., and Noller, H.F. (2006). Crystal structure of a 70S ribosome-tRNA complex reveals functional interactions and rearrangements. Cell *126*, 1065–1077.

Kucukelbir, A., Sigworth, F.J., and Tagare, H.D. (2014). Quantifying the local resolution of cryo-EM density maps. Nat. Methods *11*, 63–65.

Lancaster, L., and Noller, H.F. (2005). Involvement of 16S rRNA nucleotides G1338 and A1339 in discrimination of initiator tRNA. Mol. Cell *20*, 623–632.

Lomakin, I.B., and Steitz, T.A. (2013). The initiation of mammalian protein synthesis and mRNA scanning mechanism. Nature *500*, 307–311.

Luna, R.E., Arthanari, H., Hiraishi, H., Nanda, J., Martin-Marcos, P., Markus, M.A., Akabayov, B., Milbradt, A.G., Luna, L.E., Seo, H.C., et al. (2012). The C-terminal domain of eukaryotic initiation factor 5 promotes start codon recognition by its dynamic interplay with eIF1 and eIF2β. Cell Rep. *1*, 689–702.

Luna, R.E., Arthanari, H., Hiraishi, H., Akabayov, B., Tang, L., Cox, C., Markus, M.A., Luna, L.E., Ikeda, Y., Watanabe, R., et al. (2013). The interaction between eukaryotic initiation factor 1A and eIF5 retains eIF1 within scanning preinitiation complexes. Biochemistry *52*, 9510–9518.

Maag, D., and Lorsch, J.R. (2003). Communication between eukaryotic translation initiation factors 1 and 1A on the yeast small ribosomal subunit. J. Mol. Biol. *330*, 917–924.

Maag, D., Fekete, C.A., Gryczynski, Z., and Lorsch, J.R. (2005). A conformational change in the eukaryotic translation preinitiation complex and release of eIF1 signal recognition of the start codon. Mol. Cell *17*, 265–275.

Maag, D., Algire, M.A., and Lorsch, J.R. (2006). Communication between eukaryotic translation initiation factors 5 and 1A within the ribosomal pre-initiation complex plays a role in start site selection. J. Mol. Biol. *356*, 724–737.

Martin-Marcos, P., Nanda, J., Luna, R.E., Wagner, G., Lorsch, J.R., and Hinnebusch, A.G. (2013). β-Hairpin loop of eukaryotic initiation factor 1 (eIF1) mediates 40 S ribosome binding to regulate initiator tRNA(Met) recruitment and accuracy of AUG selection in vivo. J. Biol. Chem. *288*, 27546–27562.

Martin-Marcos, P., Nanda, J.S., Luna, R.E., Zhang, F., Saini, A.K., Cherkasova, V.A., Wagner, G., Lorsch, J.R., and Hinnebusch, A.G. (2014). Enhanced eIF1 binding to the 40S ribosome impedes conformational rearrangements of the preinitiation complex and elevates initiation accuracy. RNA *20*, 150–167.

Mindell, J.A., and Grigorieff, N. (2003). Accurate determination of local defocus and specimen tilt in electron microscopy. J. Struct. Biol. *142*, 334–347.

Mitchell, S.F., Walker, S.E., Algire, M.A., Park, E.H., Hinnebusch, A.G., and Lorsch, J.R. (2010). The 5′-7-methylguanosine cap on eukaryotic mRNAs serves both to stimulate canonical translation initiation and to block an alternative pathway. Mol. Cell *39*, 950–962.

Murshudov, G.N., Skubák, P., Lebedev, A.A., Pannu, N.S., Steiner, R.A., Nicholls, R.A., Winn, M.D., Long, F., and Vagin, A.A. (2011). REFMAC5 for the refinement of macromolecular crystal structures. Acta Crystallogr. D Biol. Crystallogr. *67*, 355–367.

Nanda, J.S., Cheung, Y.N., Takacs, J.E., Martin-Marcos, P., Saini, A.K., Hinnebusch, A.G., and Lorsch, J.R. (2009). eIF1 controls multiple steps in start codon recognition during eukaryotic translation initiation. J. Mol. Biol. *394*, 268–285.

Nanda, J.S., Saini, A.K., Muñoz, A.M., Hinnebusch, A.G., and Lorsch, J.R. (2013). Coordinated movements of eukaryotic translation initiation factors eIF1, eIF1A, and eIF5 trigger phosphate release from eIF2 in response to start codon recognition by the ribosomal preinitiation complex. J. Biol. Chem. *288*, 5316–5329.

Palam, L.R., Baird, T.D., and Wek, R.C. (2011). Phosphorylation of eIF2 facilitates ribosomal bypass of an inhibitory upstream ORF to enhance CHOP translation. J. Biol. Chem. *286*, 10939–10949.

Passmore, L.A., Schmeing, T.M., Maag, D., Applefield, D.J., Acker, M.G., Algire, M.A., Lorsch, J.R., and Ramakrishnan, V. (2007). The eukaryotic translation initiation factors eIF1 and eIF1A induce an open conformation of the 40S ribosome. Mol. Cell *26*, 41–50.

Pestova, T.V., and Kolupaeva, V.G. (2002). The roles of individual eukaryotic translation initiation factors in ribosomal scanning and initiation codon selection. Genes Dev. *16*, 2906–2922.

Pettersen, E.F., Goddard, T.D., Huang, C.C., Couch, G.S., Greenblatt, D.M., Meng, E.C., and Ferrin, T.E. (2004). UCSF Chimera—a visualization system for exploratory research and analysis. J. Comput. Chem. *25*, 1605–1612.

Pisarev, A.V., Kolupaeva, V.G., Pisareva, V.P., Merrick, W.C., Hellen, C.U., and Pestova, T.V. (2006). Specific functional interactions of nucleotides at key -3 and +4 positions flanking the initiation codon with components of the mammalian 48S translation initiation complex. Genes Dev. *20*, 624–636.

Qin, D., Abdi, N.M., and Fredrick, K. (2007). Characterization of 16S rRNA mutations that decrease the fidelity of translation initiation. RNA *13*, 2348–2355.

Rabl, J., Leibundgut, M., Ataide, S.F., Haag, A., and Ban, N. (2011). Crystal structure of the eukaryotic 40S ribosomal subunit in complex with initiation factor 1. Science *331*, 730–736.

Reibarkh, M., Yamamoto, Y., Singh, C.R., del Rio, F., Fahmy, A., Lee, B., Luna, R.E., Ii, M., Wagner, G., and Asano, K. (2008). Eukaryotic initiation factor (eIF) 1 carries two distinct eIF5-binding faces important for multifactor assembly and AUG selection. J. Biol. Chem. *283*, 1094–1103.

Rosenthal, P.B., and Henderson, R. (2003). Optimal determination of particle orientation, absolute hand, and contrast loss in single-particle electron cryomicroscopy. J. Mol. Biol. *333*, 721–745.

Roy, A., Kucukural, A., and Zhang, Y. (2010). I-TASSER: a unified platform for automated protein structure and function prediction. Nat. Protoc. *5*, 725–738.

Saini, A.K., Nanda, J.S., Lorsch, J.R., and Hinnebusch, A.G. (2010). Regulatory elements in eIF1A control the fidelity of start codon selection by modulating tRNA(i)(Met) binding to the ribosome. Genes Dev. *24*, 97–110.

Scheres, S.H. (2012). RELION: implementation of a Bayesian approach to cryo-EM structure determination. J. Struct. Biol. *180*, 519–530.

Scheres, S.H., and Chen, S. (2012). Prevention of overfitting in cryo-EM structure determination. Nat. Methods *9*, 853–854.

Schmitt, E., Panvert, M., Lazennec-Schurdevin, C., Coureux, P.D., Perez, J., Thompson, A., and Mechulam, Y. (2012). Structure of the ternary initiation complex aIF2-GDPNP-methionylated initiator tRNA. Nat. Struct. Mol. Biol. *19*, 450–454.

Selmer, M., Dunham, C.M., Murphy, F.V.T., 4th, Weixlbaumer, A., Petry, S., Kelley, A.C., Weir, J.R., and Ramakrishnan, V. (2006). Structure of the 70S ribosome complexed with mRNA and tRNA. Science *313*, 1935–1942.

Sharifulin, D., Babaylova, E., Kossinova, O., Bartuli, Y., Graifer, D., and Karpova, G. (2013). Ribosomal protein S5e is implicated in translation initiation through its interaction with the N-terminal domain of initiation factor eIF2α. ChemBioChem *14*, 2136–2143.

Shin, B.S., Kim, J.R., Walker, S.E., Dong, J., Lorsch, J.R., and Dever, T.E. (2011). Initiation factor eIF2γ promotes eIF2-GTP-Met-tRNAi(Met) ternary complex binding to the 40S ribosome. Nat. Struct. Mol. Biol. *18*, 1227–1234.

Sokabe, M., Yao, M., Sakai, N., Toya, S., and Tanaka, I. (2006). Structure of archaeal translational initiation factor 2 betagamma-GDP reveals significant conformational change of the beta-subunit and switch 1 region. Proc. Natl. Acad. Sci. USA *103*, 13016–13021.

Stolboushkina, E., Nikonov, S., Nikulin, A., Bläsi, U., Manstein, D.J., Fedorov, R., Garber, M., and Nikonov, O. (2008). Crystal structure of the intact archaeal translation initiation factor 2 demonstrates very high conformational flexibility in the alpha- and beta-subunits. J. Mol. Biol. *382*, 680–691.

Tang, G., Peng, L., Baldwin, P.R., Mann, D.S., Jiang, W., Rees, I., and Ludtke, S.J. (2007). EMAN2: an extensible image processing suite for electron microscopy. J. Struct. Biol. *157*, 38–46.

Weisser, M., Voigts-Hoffmann, F., Rabl, J., Leibundgut, M., and Ban, N. (2013). The crystal structure of the eukaryotic 40S ribosomal subunit in complex with eIF1 and eIF1A. Nat. Struct. Mol. Biol. *20*, 1015–1017.

Yu, Y., Marintchev, A., Kolupaeva, V.G., Unbehaun, A., Veryasova, T., Lai, S.C., Hong, P., Wagner, G., Hellen, C.U., and Pestova, T.V. (2009). Position of eukaryotic translation initiation factor eIF1A on the 40S ribosomal subunit mapped by directed hydroxyl radical probing. Nucleic Acids Res. *37*, 5167–5182.

A

```
18S_K.lac   UAUCUGGUUGAUCCUGCCAGUAGUCAUAUGCUUGUCUCAAAGAUUAAGCCAUGCAUGUCUAAGUAUAAGCAAUUUAUACAGUGAAACUGCGAAUGGCUCAUUAAAUCAGUUAUCGUUUAU  120
18S_S.cer   UAUCUGGUUGAUCCUGCCAGUAGUCAUAUGCUUGUCUCAAAGAUUAAGCCAUGCAUGUCUAAGUAUAAGCAAUUUAUACAGUGAAACUGCGAAUGGCUCAUUAAAUCAGUUAUCGUUUAU  120
            ************************************************************************************************************************

18S_K.lac   UUGAUAGUUCCUUUACUACAUGG-AUAUCUGUGGUAAUUCUAGAGCUAAUACAUGCUUAAAAUCUCGACCCUUUGGAAGAGAUGUAUUUAUUAGAUAAAAAAUCAAUGUCUUCGGACUCC  239
18S_S.cer   UUGAUAGUUCCUUUACUACAUGGUAUAACUGUGGUAAUUCUAGAGCUAAUACAUGCUUAAAAUCUCGACCCUUUGGAAGAGAUGUAUUUAUUAGAUAAAAAAUCAAUGUCUUCGGACUCU  240
            *********************** *** *******************************************************************************************

18S_K.lac   UUGAUGAUUCAUAAUAACUUUUCGAAUCGCAUGGCCUUGUGCUGGCGAUGGUUCAUUCAAAUUUCUGCCCUAUCAACUUUCGAUGGUAGGAUAGUGGCCUACCAUGGUUUCAACGGGUAA  359
18S_S.cer   UUGAUGAUUCAUAAUAACUUUUCGAAUCGCAUGGCCUUGUGCUGGCGAUGGUUCAUUCAAAUUUCUGCCCUAUCAACUUUCGAUGGUAGGAUAGUGGCCUACCAUGGUUUCAACGGGUAA  360
            ************************************************************************************************************************

18S_K.lac   CGGGGAAUAAGGGUUCGAUUCCGGAGAGGGAGCCUGAGAAACGGCUACCACAUCCAAGGAAGGCAGCAGGCGCGCAAAUUACCCAAUCCUAAUUCAGGGAGGUAGUGACAAUAAAUAACG  479
18S_S.cer   CGGGGAAUAAGGGUUCGAUUCCGGAGAGGGAGCCUGAGAAACGGCUACCACAUCCAAGGAAGGCAGCAGGCGCGCAAAUUACCCAAUCCUAAUUCAGGGAGGUAGUGACAAUAAAUAACG  480
            ************************************************************************************************************************

18S_K.lac   AUACAGGGCCCAUUCGGGUCUUGUAAUUGGAAUGAGUACAAUGUAAAUACCUUAACGAGGAACAACUGGAGGGCAAGUCUGGUGCCAGCAGCCGCGGUAAUUCCAGCUCCAGUAGCGUAU  599
18S_S.cer   AUACAGGGCCCAUUCGGGUCUUGUAAUUGGAAUGAGUACAAUGUAAAUACCUUAACGAGGAACAAUUGGAGGGCAAGUCUGGUGCCAGCAGCCGCGGUAAUUCCAGCUCCAAUAGCGUAU  600
            ***************************************************************** ********************************************* ********

18S_K.lac   AUUAAAGUUGUUGCAGUUAAAAAGCUCGUAGUUGAACUUUGGGUCUGGUUGUCCGGUCCGACUUUAUGUCGCGCACUGGUUUU-CAACCGGAUCUUUCCUUCUGGCUAACCUGUACUCCU  718
18S_S.cer   AUUAAAGUUGUUGCAGUUAAAAAGCUCGUAGUUGAACUUUGGGCCCGGUUGGCCGGUCCGAUUUU--UUCGUGUACUGGAUUUCCAACGGGGCCUUUCCUUCUGGCUAACCUUGAGUCCU  718
            ******************************************* * ***** ********* ***   *** * ***** *** **** **  *******************  * ****

18S_K.lac   UGUGGGUGCAGGCGAACCAGGACUUUUACUUUGAAAAAAUUAGAGUGUUCAAAGCAGGCGAA-AGCUCGAAUAUAUUAGCAUGGAAUAAUGGAAUAGGACGUUUGGUUCUAUUUUGUUGG  837
18S_S.cer   UGUGGCUCUUGGCGAACCAGGACUUUUACUUUGAAAAAAUUAGAGUGUUCAAAGCAGGCGUAUUGCUCGAAUAUAUUAGCAUGGAAUAAUAGAAUAGGACGUUUGGUUCUAUUUUGUUGG  838
            ***** *   ************************************************** *  ************************ *******************************

18S_K.lac   UUUCUAGGACCAUCGUAAUGAUUAAUAGGGACGGUCGGGGGCAUCAGUAUUCAAUUGUCAGAGGUGAAAUUCUUGGAUUUAUUGAAGACUAACUACUGCGAAAGCAUUUGCCAAGGACGU  957
18S_S.cer   UUUCUAGGACCAUCGUAAUGAUUAAUAGGGACGGUCGGGGGCAUCAGUAUUCAAUUGUCAGAGGUGAAAUUCUUGGAUUUAUUGAAGACUAACUACUGCGAAAGCAUUUGCCAAGGACGU  958
            ************************************************************************************************************************

18S_K.lac   UUUCAUUAAUCAAGAACGAAAGUUAGGGGAUCGAAGAUGAUCAGAUACCGUCGUAGUCUUAACCAUAAACUAUGCCGACUAGGGAUCGGGUGGUGUUUUUCUUAUGACCCACUCGGCACC  1077
18S_S.cer   UUUCAUUAAUCAAGAACGAAAGUUAGGGGAUCGAAGAUGAUCAGAUACCGUCGUAGUCUUAACCAUAAACUAUGCCGACUAGGGAUCGGGUGGUGUUUUUUUAAUGACCCACUCGGCACC  1078
            **************************************************************************************************** * *****************

18S_K.lac   UUACGAGAAAUCAAAGUCUUUGGGUUCUGGGGGGAGUAUGGUCGCAAGGCUGAAACUUAAAGGAAUUGACGGAAGGGCACCACCAGGAGUGGAGCCUGCGGCUUAAUUUGACUCAACACG  1197
18S_S.cer   UUACGAGAAAUCAAAGUCUUUGGGUUCUGGGGGGAGUAUGGUCGCAAGGCUGAAACUUAAAGGAAUUGACGGAAGGGCACCACCAGGAGUGGAGCCUGCGGCUUAAUUUGACUCAACACG  1198
            ************************************************************************************************************************

18S_K.lac   GGGAAACUCACCAGGUCCAGACACAAUAAGGAUUGACAGAUUGAGAGCUCUUUCUUGAUUUUGUGGGUGGUGGUGCAUGGCCGUUCUUAGUUGGUGGAGUGAUUUGUCUGCUUAAUUGCG  1317
18S_S.cer   GGGAAACUCACCAGGUCCAGACACAAUAAGGAUUGACAGAUUGAGAGCUCUUUCUUGAUUUUGUGGGUGGUGGUGCAUGGCCGUUCUUAGUUGGUGGAGUGAUUUGUCUGCUUAAUUGCG  1318
            ************************************************************************************************************************

18S_K.lac   AUAACGAACGAGACCUUAACCUACUAAAUAGGGUUGCUGGCACUUGCCGGUUGA-CUCUUCUUAGAGGGACUAUCGGUUUCAAGCCGAUGGAAGUUUGAGGCAAUAACAGGUCUGUGAUG  1436
18S_S.cer   AUAACGAACGAGACCUUAACCUACUAAAUAGUGGUGCUAGCAUUUGCUGGUUAUCCACUUCUUAGAGGGACUAUCGGUUUCAAGCCGAUGGAAGUUUGAGGCAAUAACAGGUCUGUGAUG  1438
            ******************************* * **** *** **** ****   * ***************************************************************

18S_K.lac   CCCUUAGACGUUCUGGGCCGCACGCGCGCUACACUGACGGAGCCAGCGAGUACAACCUUGGCCGAGAGGUCUGGGUAAUCUUGUGAAACUCCGUCGUGCUGGGGAUAGAGCAUUGUAAUU  1556
18S_S.cer   CCCUUAGACGUUCUGGGCCGCACGCGCGCUACACUGACGGAGCCAGCGAGUCUAACCUUGGCCGAGAGGUCUUGGUAAUCUUGUGAAACUCCGUCGUGCUGGGGAUAGAGCAUUGUAAUU  1558
            ***************************************************  ******************* ***********************************************

18S_K.lac   AUUGCUCUUCAACGAGGAAUUCCUAGUAAGCGCAAGUCAUCAGCUUGCGUUGAUUACGUCCCUGCCCUUUGUACACACCGCCCGUCGCUAGUACCGAUUGAAUGGCUUAGUGAGGCCUCA  1676
18S_S.cer   AUUGCUCUUCAACGAGGAAUUCCUAGUAAGCGCAAGUCAUCAGCUUGCGUUGAUUACGUCCCUGCCCUUUGUACACACCGCCCGUCGCUAGUACCGAUUGAAUGGCUUAGUGAGGCCUCA  1678
            ************************************************************************************************************************

18S_K.lac   GGAUUUGCUUAGAGAAGGGGGCAACUCCAUCUCAGAGCGAAGAAUCUGGUCAAACUUGGUCAUUUAGAGGAACUAAAAGUCGUAACAAGGUUUCCGUAGGUGAACCUGCGGAAGGAUCAU  1796
18S_S.cer   GGAUCUGCUUAGAGAAGGGGGCAACUCCAUCUCAGAGCGGAGAAUUUGGACAAACUUGGUCAUUUAGAGGAACUAAAAGUCGUAACAAGGUUUCCGUAGGUGAACCUGCGGAAGGAUCAU  1798
            **** ********************************** ***** *** **********************************************************************

18S_K.lac   UA  1798
18S_S.cer   UA  1800
            **
```

B

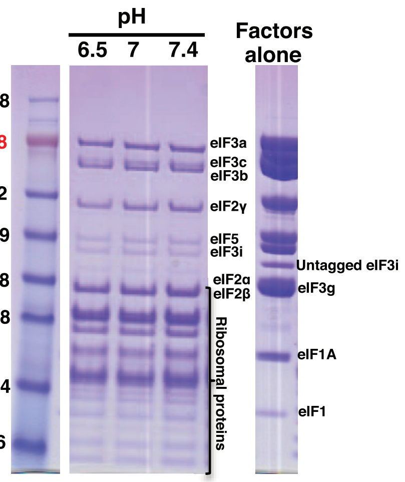

(legend on next page)

Figure S1. *K lactis* rRNA Sequence and PIC Formation Using *K. lactis* 40S and *S. cerevisiae* Initiation Factors, Related to Figure 1

(A) Alignment of 18S rRNA sequences from *K. lactis* and *S. cerevisiae*.

(B) SDS-PAGE separation of the fraction corresponding to 48S from 10%–30% sucrose density gradient ultracentrifugation formed using 40S subunits from *K. lactis* and initiation factors from *S. cerevisiae* at different pH values. All the protein factors migrating more slowly than ribosomal proteins are easily identified in the gel, including those (eIF3 subunits and eIF5) not clearly visible in the present cryoEM reconstruction. The positions of ligands and standard protein markers are shown in separate lanes on the right and left extremes, respectively. All lanes belong to the one gel and the adjoining lanes have been removed for clarity.

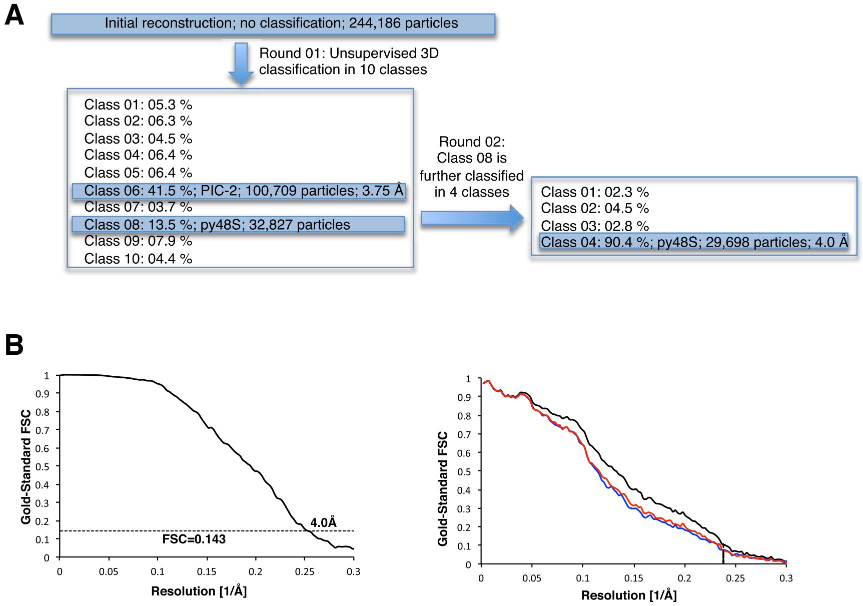

Figure S2. Electron cryoEM Data Processing and Map Quality, Related to Figure 1

(A) Maximum-likelihood 3D classification scheme (See Experimental Procedures).

(B) At the left, Gold-standard Fourier Shell Correlation (FSC) curve for the py48S structure. At the right, FSC_{work} curves (red) corresponding to the refined model versus the half-map it was refined against, and FSC_{test} curves (blue), i.e., those calculated between the refined atomic model and the other half-map. The black curve shows the FSC curve between a reconstruction from all particles and the model refined against the map.

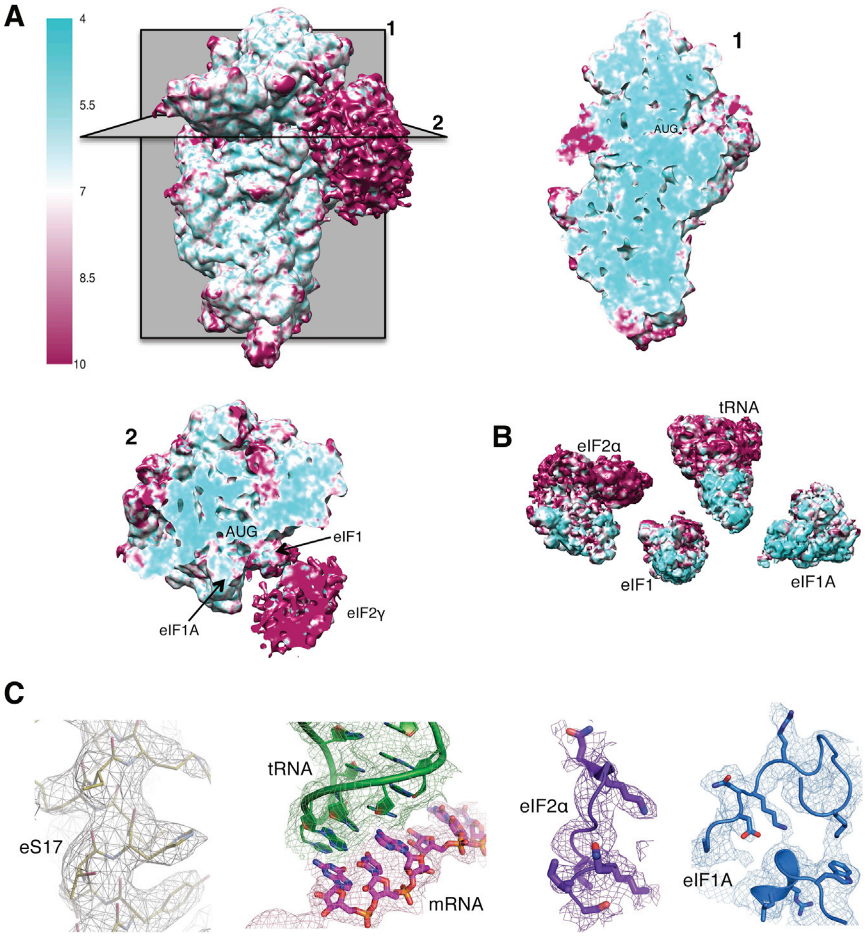

Figure S3. Local Resolution Features of py48S Complex, Related to Figure 1

(A) Surface (left) and cross-sections along the mRNA channel in two different planes of an 8 Å-filtered map, colored according to local resolution (See Experimental Procedures).

(B) Surface representations of the different ligands, viewed from its ribosome interacting surface and colored according to local resolution (eIF1A-4.6 Å; eIF1-6.8 Å; eIF2α-6.9 Å; tRNA-5.9 Å; ASL of $tRNA_i$ and mRNA from −4 to +4 position - 4.7 Å).

(C) High-resolution features for protein eS17, $tRNA_i$, mRNA, eIF2α and eIF1A at 4.0 Å resolution.

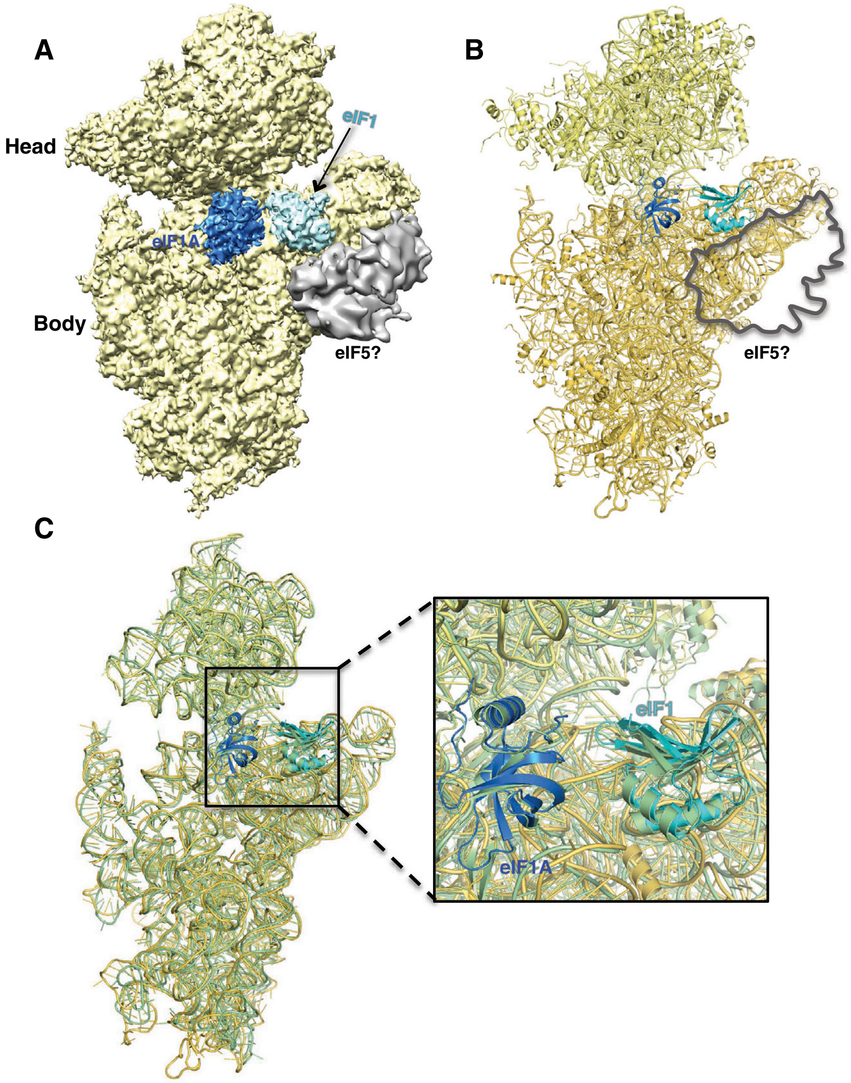

Figure S4. Cryo-EM Structure of PIC-2, Related to Figure 1

(A) Map reconstruction of PIC-2 sharpened at 3.8 Å. The map is segmented and colored in yellow (40S subunit), blue (eIF1A), cyan (eIF1). An unassigned density in contact with eIF1 is colored gray and low pass filtered to 8 Å, putatively assigned to eIF5.

(B) Atomic model for PIC-2. The complex is oriented and colored as in A, except for the head and body of 40S, which are displayed in different shades of yellow. A profile of the aforementioned unassigned density is also shown.

(C) Superposition of PIC-2 and 40S·eIF1·eIF1A from *T. thermophila* (from 4BPE; colored in green). Only rRNA, eIF1 and eIF1A are shown. A zoomed-in view, including also the ribosomal proteins, of the A and P sites where eIF1A and eIF1 bind is shown (inset).

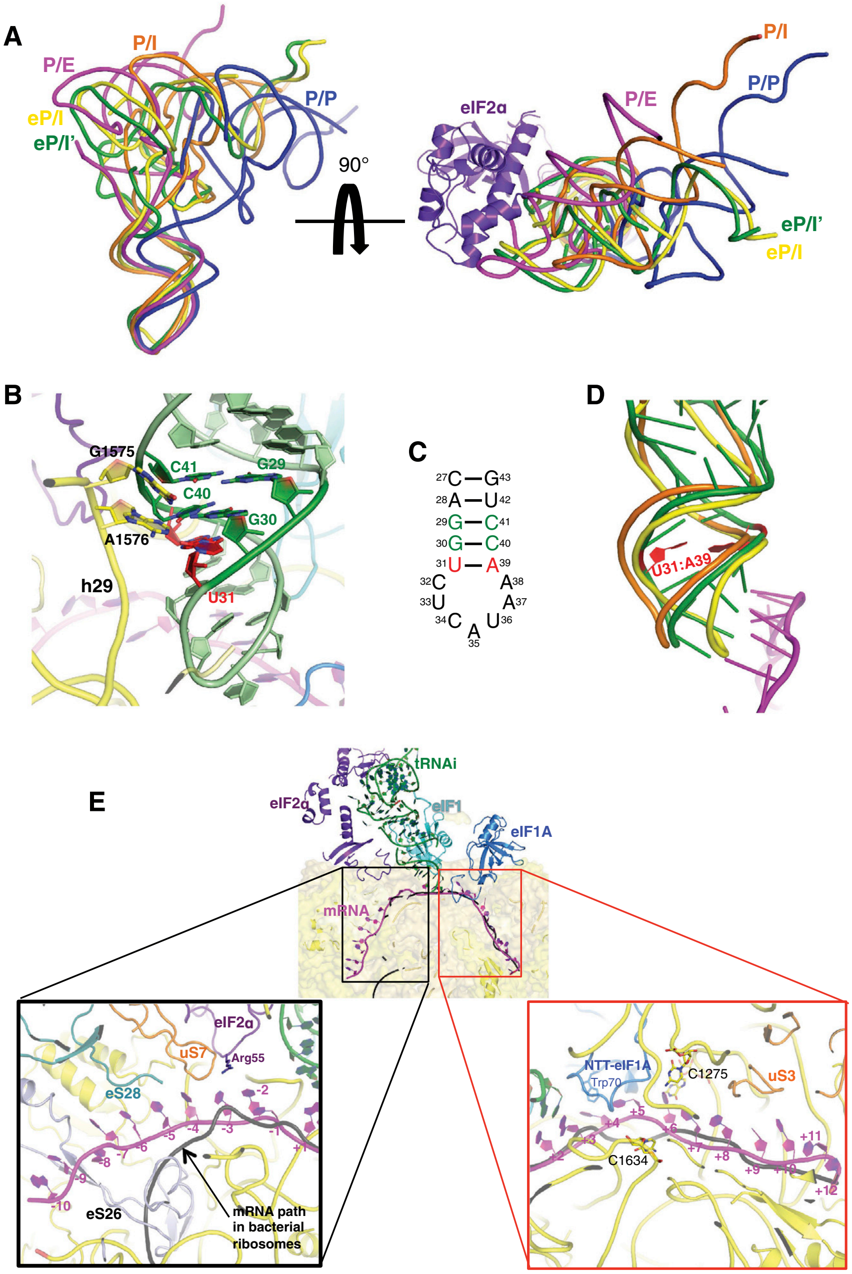

Figure S5. eP/I′ tRNA$_i$ and mRNA Path, Related to Figure 2

(A) Two different views of the superimposition of tRNAs from different complexes. Conformation of tRNA$_i$ from py48S (green) is similar to that described for pm43S complex [eP/I, yellow; (Hashem et al., 2013)], but different to canonical P/P site (from 2J00; blue), P/E (from 4JUW; magenta), or P/I conformations (from pm48S PIC, in orange, from 4KZZ). In the view at the right, eIF2α is also shown to highlight the orientation of the elbow of the tRNA$_i$ toward the E site.

(B) Recognition of conserved G:C base pairs in the ASL of tRNA$_i$ by rRNA bases G1575 and A1576.

(C). Sequence of the ASL of the tRNA$_i$ used in this study.

(D) ASL conformational change upon codon-anticodon interaction. tRNA$_i$ from py48S (green), pm48S (orange) and pm43S (yellow) are compared. The mutation U31:A39 in tRNA$_i$ used in this study is highlighted in red.

(E) Cross-section of the 40S subunit along the mRNA path (in magenta), viewed from the top of the 40S subunit. The bacterial mRNA path is also shown for comparison (from 2HGP, semitransparent black representation). (Black box) mRNA at the exit channel (from −1 to −10). eIF2α subunit is colored violet, and mRNA-interacting ribosomal proteins, uS7, eS26 and eS28 are colored orange, blue-white and teal, respectively. (Red box) mRNA at the entry channel (from +2 to +12). rRNA bases interacting with mRNA at the A-site are shown in sticks representation. uS3 is colored orange.

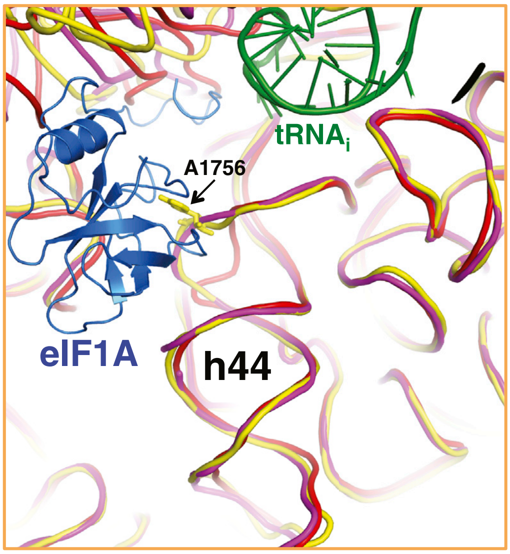

Figure S6. Relative Orientation of eIF1A and h44, Showing A1756 Flipped Out to Interact with eIF1A, Related to Figure 4

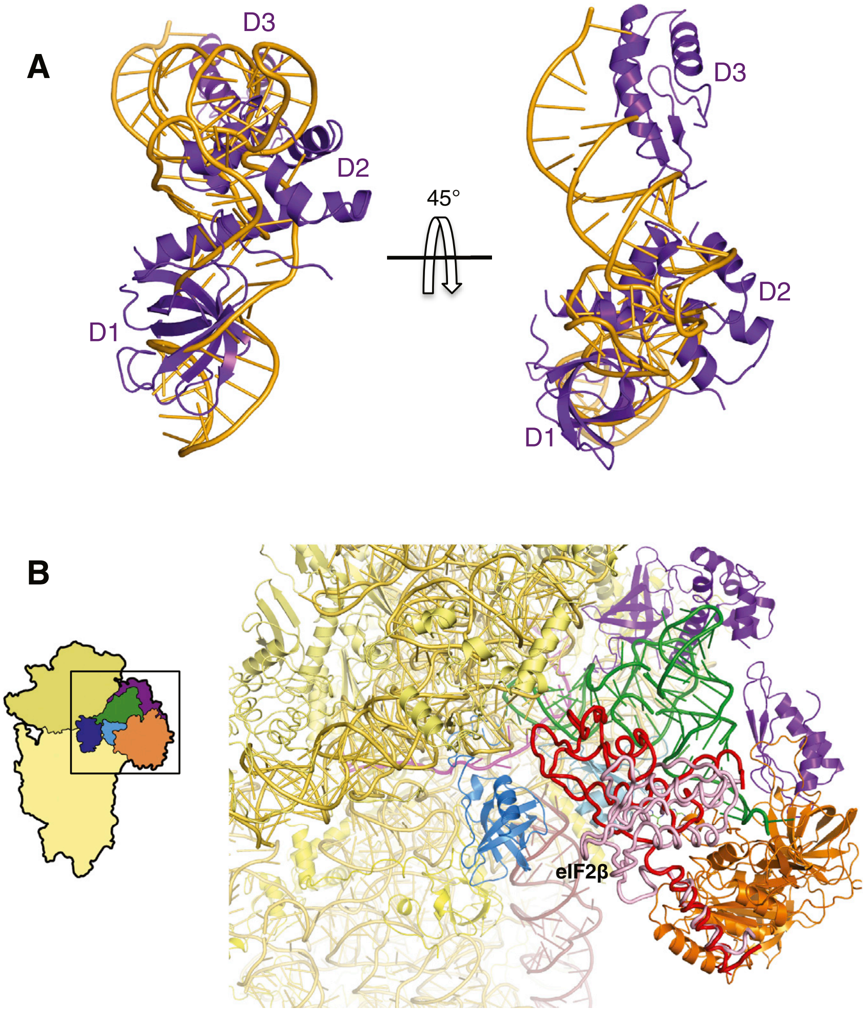

Figure S7. eIF2α and E-site tRNA and Possible Locations of eIF2β, Related to Figure 6

(A) eIF2α mimics E-site tRNA. An E-site tRNA (from 2J00) and eIF2α are shown superimposed relative to their positions in the E-site.

(B) Possible locations of eIF2β in the complex. The β subunits from two different archaeal aIF2 crystal structures (from 3CW2, in red and from 2D74, in pink) have been modeled in the py48S PIC by superimposing the aIF2γ subunit onto our fitted eIF2γ subunit.
